# Shape Invariant Coding of Motion Direction in Somatosensory Cortex

**DOI:** 10.1371/journal.pbio.1000305

**Published:** 2010-02-02

**Authors:** Yu-Cheng Pei, Steven S. Hsiao, James C. Craig, Sliman J. Bensmaia

**Affiliations:** 1 Zanvyl Krieger Mind/Brain Institute, Johns Hopkins University, Baltimore, Maryland, United States of America; 2 Solomon H. Snyder Department of Neuroscience, Johns Hopkins University, Baltimore, Maryland, United States of America; 3 Department of Physical Medicine and Rehabilitation, Chang Gung Memorial Hospital and Chang Gung University, Taiwan; 4 Psychological and Brain Sciences, Indiana University, Bloomington, Indiana, United States of America; 5 Department of Organismal Biology and Anatomy, University of Chicago, Chicago, Illinois, United States of America; Vanderbilt University, United States of America

## Abstract

Invariant representations of stimulus features are thought to play an important role in producing stable percepts of objects. In the present study, we assess the invariance of neural representations of tactile motion direction with respect to other stimulus properties. To this end, we record the responses evoked in individual neurons in somatosensory cortex of primates, including areas 3b, 1, and 2, by three types of motion stimuli, namely scanned bars and dot patterns, and random dot displays, presented to the fingertips of macaque monkeys. We identify a population of neurons in area 1 that is highly sensitive to the direction of stimulus motion and whose motion signals are invariant across stimulus types and conditions. The motion signals conveyed by individual neurons in area 1 can account for the ability of human observers to discriminate the direction of motion of these stimuli, as measured in paired psychophysical experiments. We conclude that area 1 contains a robust representation of motion and discuss similarities in the neural mechanisms of visual and tactile motion processing.

## Abstract 2

A subpopulation of neurons in primate somatosensory cortex signal the direction in which objects move across the skin of the fingertips.

## Author Summary

When we physically interact with an object, our hands convey information about the shape of the object, its texture, its compliance, and its thermal properties. This information allows us to manipulate tools and to recognize objects based on tactile exploration alone. One of the hallmarks of tactile object recognition is that it involves movement between the skin and the object. In this study, we investigate how the direction in which objects move relative to the skin is represented in the brain. Specifically, we scan a variety of stimuli, including bars and dot patterns, across the fingers of non-human primates while recording the evoked neuronal activity. We find that a population of neurons in somatosensory cortex encodes the direction of moving stimuli regardless of the shape of the stimuli, the speed at which they are scanned across the skin, or the force with which they contact the skin. We show that these neurons can account for our ability to perceive the direction of motion of tactile stimuli.

## Introduction

In both vision and touch, information about form and motion is inferred from a spatio-temporal pattern of activation across a two-dimensional sensory sheet (the retina and the skin). The early stages of form processing have been shown to be similar in these two modalities in that both involve decomposing the stimulus into a set of local oriented contours [1]–[3]. Furthermore, the tactile integration of local motion cues has been shown, in psychophysical experiments, to be analogous to its visual counterpart [4] and the visual and tactile perception of motion have been shown to interact (see e.g. [5],[6]). In previous studies of motion processing in primary somatosensory cortex (S1), a population of neurons has been identified whose responses are modulated by the direction of stimulus motion [7]–[10]. Directionally sensitive neurons have been found in three areas of what is traditionally considered S1, namely 3b, 1, and 2. The question remains how representations of motion are elaborated in these three cortical areas.

**Figure 1:**
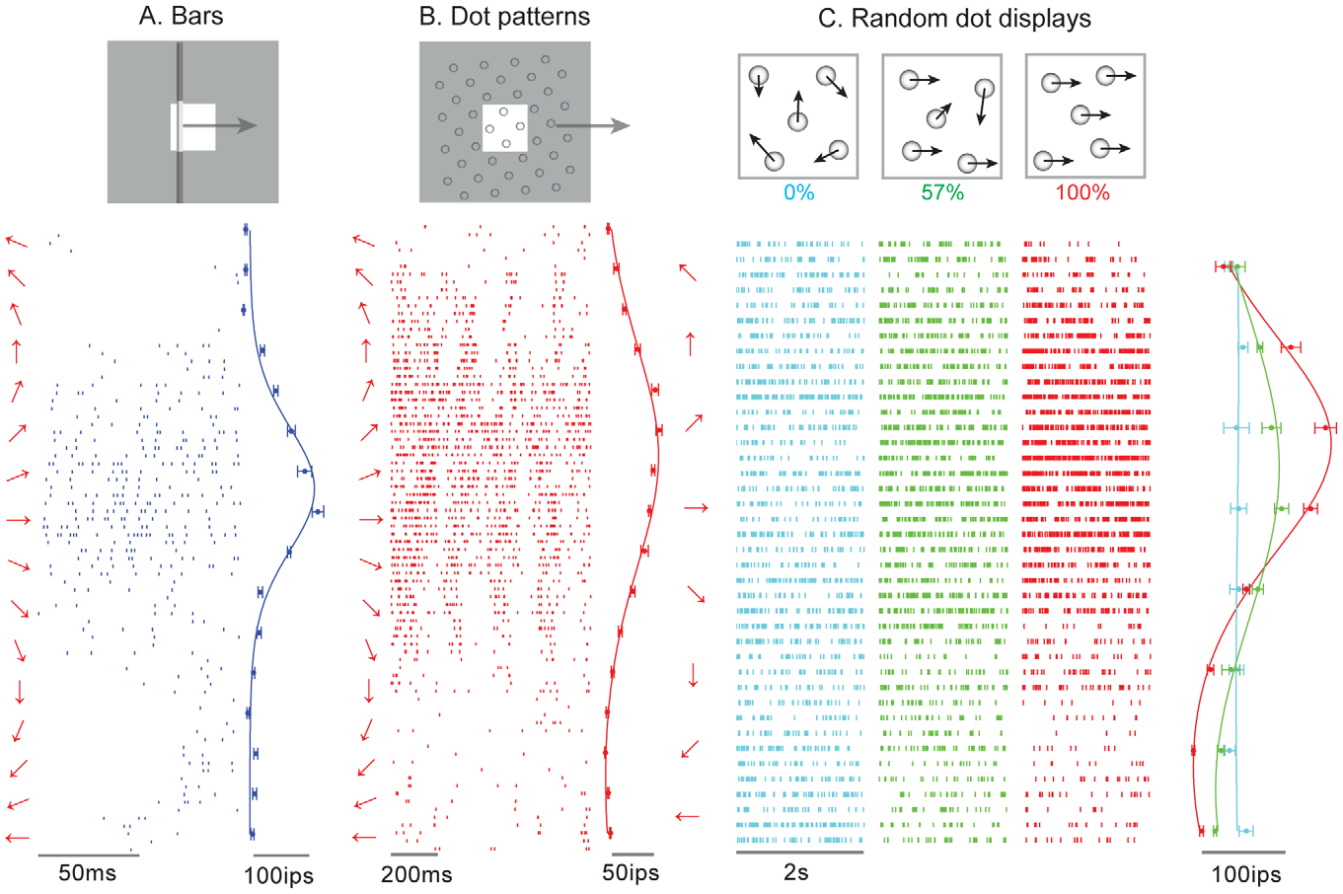
Responses of a neuron in area 1 to (A) bars (DI = 0.42), (B) dot patterns (DI = 0.54), and (C) random dot displays (DI = 0.52 at 100% coherence) presented to the monkey's fingertip. The stimuli are illustrated as insets at the top: for bars and dot patterns, the white square shows the area (1 cm×1 cm) across which the stimuli are scanned; the gray region illustrates the stimulus extending outside of the stimulation area. The left of each raster is the direction of motion of the stimulus. To the right of each raster is the mean firing rate evoked by stimuli moving in each direction. This neuron produced the most robust response to stimuli moving at approximately 20°, regardless of whether the stimuli were bars, dot patterns, or random dot displays. For random dot displays, the direction tuning increased dramatically with increases in the motion coherence (we show responses at only three levels of coherence for the sake of clarity). The “burstiness” of the response to dot patterns likely reflects individual dots moving across the neuron's hotspot.

In the present study, we investigate the representation of motion in S1 using experimental paradigms inspired by vision research. To that end, we deliver three types of motion stimuli—bars, dot patterns, and random dot displays—to the fingertips of Rhesus macaques while recording the responses these stimuli evoke in neurons in areas 3b, 1, and 2. Stimuli are delivered using a 400-probe stimulator, the tactile analogue of a visual monitor [11]. Probes are indented into the skin in spatio-temporal sequences, analogously to pixels on a monitor, to produce verisimilar percepts of shape and motion. Because the spacing between adjacent probes is shorter than that between adjacent mechanoreceptors in the skin, the inherent pixelation of the array is not felt. The bars and dot patterns are scanned across the receptive field (RF) in each of 16 directions ranging from 0° to 360°. The random dot displays are the tactile analogues of stimuli that have been widely used in studies of visual motion [12],[13]. In the tactile version, a set of five hemispheric dots move across the skin surface (see inset of Figure 1C); the degree to which the dots move in a coherent direction can be varied. At one extreme (0% coherence), the direction of motion of each dot at each point in time is determined randomly. In this condition, the display cannot and does not yield any holistic percept of motion direction. At the other extreme (100% coherence), all the dots move in the same direction, and the display yields a robust percept of motion direction. At intermediate levels of coherence, the probability that individual dots move in the prescribed direction is set (at a level determined by the motion coherence) between 0% (chance) and 100%. In a paired psychophysical study, we measured the ability of human subjects to discriminate the direction of motion of these same stimuli presented to their left index fingertips.

The objective of the present study was to ascertain (1) whether a population of neurons in areas 3b, 1, and 2 conveyed motion information that was invariant relative to the spatial properties of the stimulus (i.e., its two-dimensional form); (2) whether the direction signal was modulated by motion coherence (in the case of random dot displays) as has been found for neurons in area MT; and (3) whether the motion signal conveyed by a subpopulation of neurons in areas 3b, 1, and 2 could account for psychophysical performance across paradigms and stimulus types.

## Results/Discussion

We recorded the responses of 20 SA1 and 11 RA afferents, and 92, 148, and 37 neurons in areas 3b, 1, and 2, respectively (peripheral afferents were tested only with scanned bars; only a subset of cortical neurons was tested with all stimuli) (see Table 1). Figure 1 shows the responses of a neuron in area 1 to (A) scanned bars, (B) dot patterns and (C) random dot displays varying in coherence. The neuron responded most strongly when stimuli moved medial to lateral relative to the midline with a slight proximal to distal slant (preferred direction = 20°). Importantly, its preferred direction was approximately the same across stimulus types, demonstrating that this neuron conveys information about stimulus direction that is invariant with respect to spatial form. Furthermore, the neuron's responses to all the random dot displays were equal when their coherence was 0% (cyan rasters and tuning curve in C) but direction tuning emerged and then sharpened as the motion coherence increased.

**Table 1:** Fraction and percentage of neurons whose responses were significantly tuned to each type of stimulus.

	Bars		Dot Patterns		Random Dot Displays	
	Fraction	%	Fraction	%	Fraction	%
Area 3b	28/92	30	27/61	44	3/16	19
Area 1	85/148	57	80/136	59	27/42	42
Area 2	20/37	54	6/35	18	4/26	15

**Figure 2:**
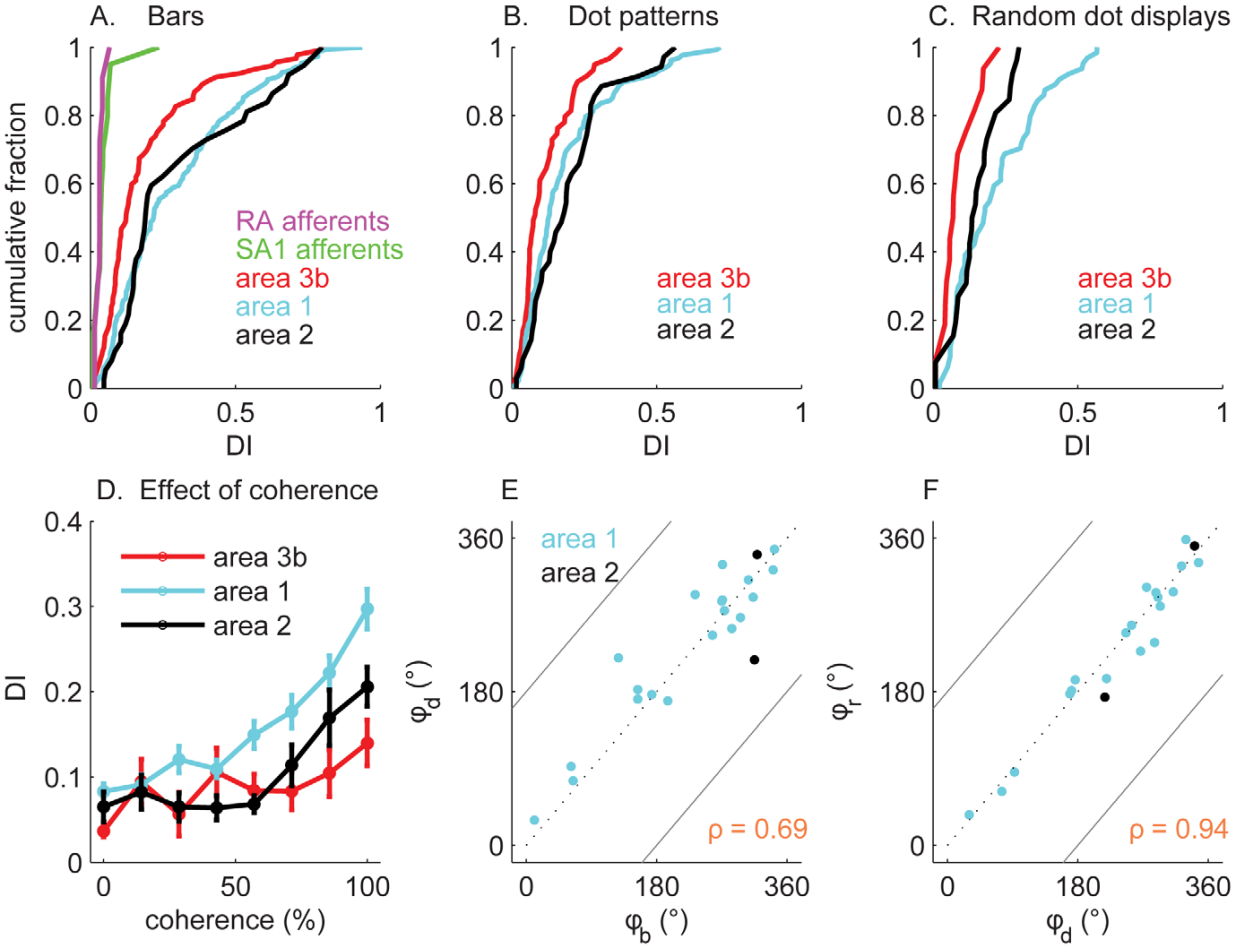
Direction tuning in primary somatosensory cortex. (A) Distribution of the direction tuning index, DI, derived from the responses of peripheral afferents and of neurons in areas 3b, 1, and 2 to bars scanned across the fingertip. The responses of individual afferents were not sensitive to the direction of stimulus motion, whereas the responses of many cortical neurons were. The DIs obtained from neurons in area 1 (mean ± s.e.m.: 0.25±0.02) were significantly higher than those obtained from neurons in area 3b (s.e.m.: 0.19±0.02) (*t*(178) = 2.1, *p*<0.05). (B) Cumulative distributions of DI obtained from dot patterns scanned across the fingertip. Direction tuning was stronger in area 1 (0.18±0.02) than it was in area 3b (0.13±0.01) (*t*(138) = 2.1, *p*<0.05). Furthermore, tuning was weaker for dot patterns than for bars. (C) Cumulative distributions of DI obtained from random dot displays delivered to the fingertip. Direction tuning was stronger in area 1 (0.21±0.03) than it was in area 3b (0.11±0.02) (*t*(32) = 1.9, *p* = 0.07). (D) DI as a function of the motion coherence of the random dot displays for neurons in areas 3b (red), 1 (cyan), and 2 (black). The direction tuning of area 1 neurons increased monotonically with motion coherence, whereas that of area 3b neurons did not. Area 2 neurons exhibited tuned responses only when the motion signal was highly coherent (>70%). (E) Preferred direction measured from responses to dot patterns (*ϕ_d_*) versus preferred direction measured from responses to bars (*ϕ_b_*) for neurons that yielded a significant DI for bars, dot patterns, and random dot displays (no neurons in area 3b and only two neurons in area 2 met this criterion). The circular correlation between *ϕ_d_* and *ϕ_b_* was 0.69 overall (*p*<0.01). 0° denotes left to right motion, 90° proximal to distal, 180° right to left, and 270° distal to proximal. (F). Preferred direction measured from responses to random dot displays at 100% coherence (*ϕ_r_*) versus that measured from responses to dot patterns (*ϕ_d_*) for neurons significantly tuned for all three types of stimuli. The circular correlation between *ϕ_d_* and *ϕ_r_* was 0.94 (*p*<0.01). Most neurons yielded comparable preferred directions to the two types of stimuli.

To quantify the strength of tuning, we derived a direction selectivity index (DI) (see Materials and Methods) that increased from 0 to 1 as tuning strength increased. Figure 2A shows the cumulative histogram of DI obtained from the responses of peripheral afferents and neurons in areas 3b, 1, and 2 to scanned bars. Responses of individual SA1 and RA afferents were not tuned for direction as evidenced by the fact that they yielded DIs near zero (Figure 2A; also see Figures S2 and S3). In contrast, tuning for direction is evident at the earliest stage of cortical processing, namely in area 3b, which comprised a large proportion of neurons that were sensitive to the direction of motion of scanned bars. Direction tuning for bars was greater in areas 1 and 2, which contained a much larger proportion of neurons that exhibited strong direction tuning than did area 3b (Table 1). Although direction tuning in responses to dot patterns was present in areas 3b and 2, it was stronger for neurons in area 1 (Figure 2B, Table 1) (note that, although the numerical value of DI derived from responses to dot patterns tended to be higher for neurons in area 2 than in area 3b, many of the DIs derived from area 2 responses were not statistically reliable, as shown in Table 1). Finally, neurons in areas 3b and 2 exhibited only weak direction tuning in their responses to random dot displays at 100% coherence (Figure 2C), whereas the responses of a large proportion of area 1 neurons were strongly tuned for direction. Area 2 neurons exhibited particularly weak direction tuning to dot patterns, suggesting that these neurons are sensitive to edges; indeed, a large proportion of area 2 neurons are orientation selective (unpublished data). Despite the fact that area 2 is higher in the somatosensory pathway than area 1, it seems that the latter comprises a more robust representation of direction of motion than does the former.

Next, we examined the effect of motion coherence on direction tuning in direction-sensitive neurons. We found that the increase in tuning strength was marginal for neurons in area 3b, whereas it was substantial for neurons in area 1 (Figure 2D). The tuning strength of area 2 neurons exhibited an intermediate dependence on motion coherence, and direction tuning for these neurons only emerged at high levels of coherence (>70%).

We then wished to ascertain (1) whether individual neurons conveyed information about direction across stimulus types and (2) whether the direction signal conveyed by those neurons remained unchanged as the stimulus type changed. To this end, we identified a population of neurons that were significantly tuned for bars, dot patterns, and random dot displays. We found that no neurons in area 3b and 8% of the neurons (2 of 25 neurons) in area 2 that were tested with all three types of patterns exhibited significantly direction-tuned responses to all three stimulus types. In contrast, 30% (14 of 42) of the neurons in area 1 exhibited significant direction tuning independent of stimulus type, with a large majority having the same preferred direction for all three stimulus types (Figure 2E and 2F).

**Figure 3:**
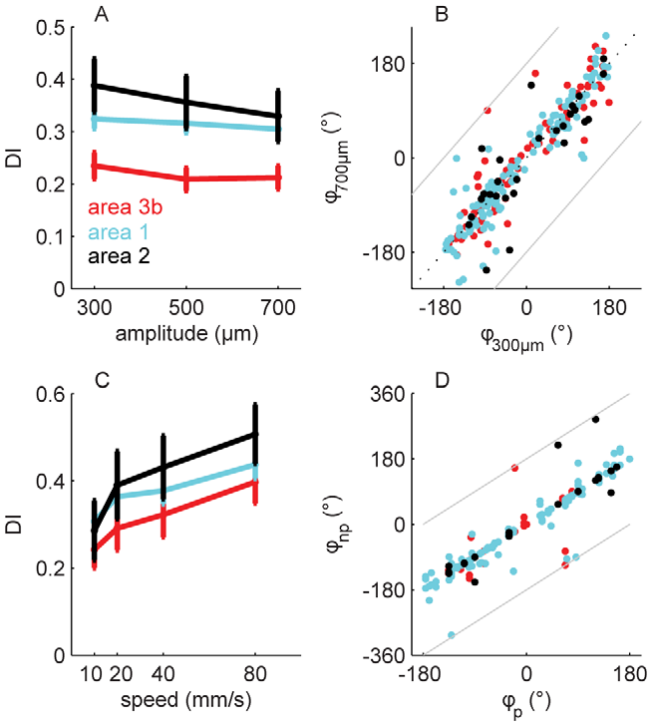
Invariance of direction tuning with respect to amplitude (A,B) and speed (C,D) for bars scanned across the fingertip. (A) Direction selectivity index (DI) as a function of amplitude. (B) Preferred direction obtained from bars of amplitude 700 µm (*ϕ_700µm_*) versus that obtained from bars of amplitude 300 µm (*ϕ_300µm_*). (C) DI as a function of scanning speed. Preferred direction at a non-preferred speed (*ϕ_np_*) versus that at the preferred speed (*ϕ_p_*) for every combination for which direction tuning was significant. Different neurons were more strongly direction-tuned at different speeds with a tendency for neurons to be more strongly tuned at higher speeds (see Figure S4). For the neurons whose direction tuning changed as a function of scanning speed, the increase in tuning could be attributed to an increased firing rate in the preferred direction, to a decreased firing rate in the anti-preferred direction, or to both. A similar modulation of tuning strength by speed is also observed in primary visual cortex.

The direction signal conveyed by these neurons was also largely insensitive to changes in the stimulus amplitude (i.e., the indentation amplitude), or scanning speed over a wide range of behaviorally relevant amplitudes and speeds. The strength of direction tuning was not significantly modulated by stimulus amplitude (Figure 3A; *F*(2,705) = 0.4, *p*>0.6). Furthermore, with few exceptions, the preferred direction was the same across stimulus amplitudes (Figure 3B, 84% of direction selective neurons yielded preferred directions that differed by less than 45° across the two amplitudes, in contrast to afferents; see Figure S3). Similarly, while strength of tuning increased slightly but significantly with scanning speed across the population (Figure 3C; *F*(3,540) = 7.3, *p*<.01), the strength of direction tuning of individual neurons exhibited a wide variety of relationships with scanning speed (Figure S4), as did the strength of their responses (unpublished data). Importantly, the preferred direction of individual neurons was consistent across speeds (Figure 3D).

**Figure 4:**
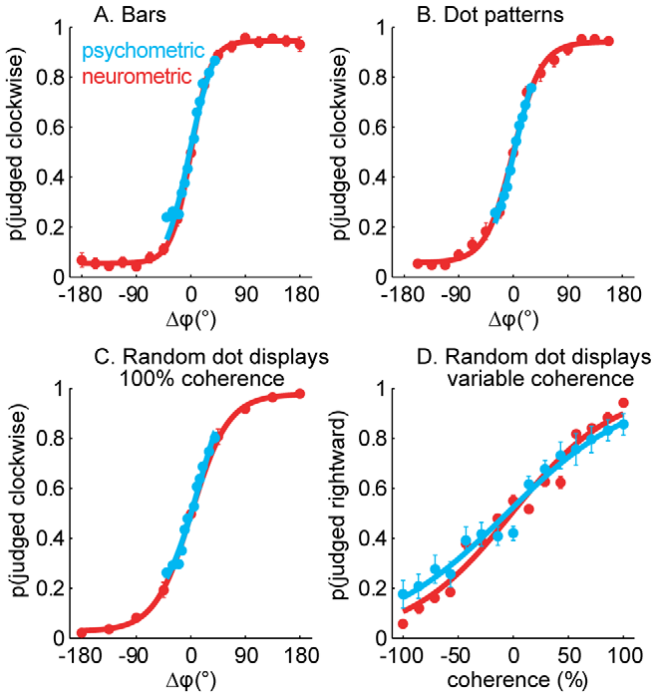
Psychometric functions and neurometric functions obtained from the clockwise-counterclockwise discrimination task with (A) bars, (B) dot patterns, and (C) random dot displays (with 100% coherence) delivered to the fingertip. The neurometric functions were derived from the responses of the neurons that exhibited significant direction tuning to bars, dot patterns, and random dot displays. A positive Δϕ denotes a clockwise rotation. As can be seen, the direction information carried by these neurons can account for the perceived direction of the stimuli. (D) Psychometric and neurometric functions obtained for the left-right discrimination task with random dot displays varying in coherence. Again, the responses of directionally selective area 1 neurons could account for perception.

As shown above, a subpopulation of neurons in areas 3b, 1, and 2 conveys significant information about direction of motion for each stimulus type (Figure 2). Can the responses of these neurons account for our ability to discriminate direction of motion? We derived psychometric functions from clockwise-counterclockwise judgments obtained from human subjects and compared them to analogous “judgments” derived from the responses of individual neurons. Specifically, we used an ideal observer analysis to determine the extent to which stimuli moving in different directions could be discriminated on the basis of the responses these evoked in individual neurons. We found that the responses of the most direction-selective neurons in area 1 could account for psychophysical performance (Figure 4). (We carried out this analysis using data only from neurons that were significantly direction selective for bars, dot patterns, and random dot displays. No neurons in area 3b and only 8% of neurons in area 2 met our selection criterion; see above.). Indeed, the direction of motion of bars, dot patterns, and random dots could be distinguished on the basis of the responses of a subpopulation of neurons in area 1 with the same accuracy as that observed in human psychophysical experiments (Figure 4A–C, also see Figure S5). Furthermore, the sensitivity of the direction signal to motion coherence mirrored that of human subjects (Figure 4D). Our results are therefore consistent with the hypothesis that area 1 comprises a population of neurons whose responses underlie our ability to perceive the direction of tactile motion. However, the neuronal and behavioral data were obtained from different species; this hypothesis could be tested in future experiments by assessing whether electrically stimulating clusters of direction selective neurons systematically affects the animal's performance in a direction discrimination task [14] or by ascertaining whether the responses of direction selective neurons are predictive of a monkey's behavior [15],[16].

**Figure 5:**
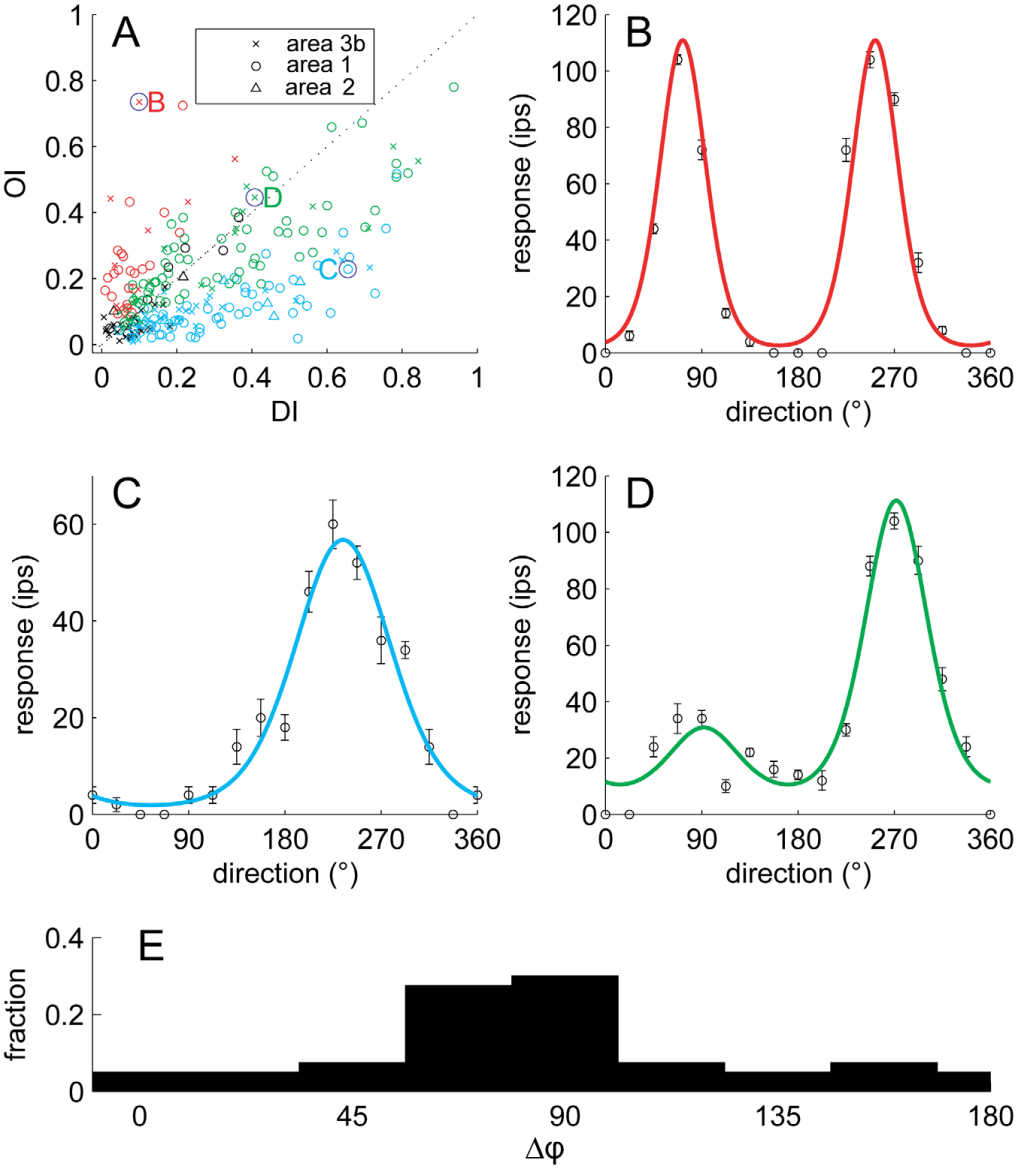
Orientation sensitivity and direction sensitivity. (A) Orientation selectivity index (OI) versus direction selectivity index (DI) for neurons in areas 3b (crosses), 1 (circles), and 2 (triangles). Red symbols represent neurons that yielded significant OIs, blue symbols represent neurons that yielded significant DIs, and green symbols represent neurons for which both indices were significant; black symbols represent neurons for which neither index was significant. (B) Responses of a neuron that yielded a high OI and a low DI (marked as a *B* in the upper left quadrant of the scatterplot); red trace is a fitted von Mises function (circular Gaussian); (C) responses of a neuron that yielded a high DI and a low OI (marked as a *C* in the lower right quadrant); blue trace is a fitted von Mises function; (D) responses of a neuron that yielded intermediate values for both the OI and the DI (marked as a *D* around the center); green trace is the product of two von Mises functions; (E) Distribution of the angular difference between the preferred direction (measured from dot patterns) and the preferred orientation (measured from indented bars) of neurons in area 1. For orientation, 90° and 270° denote bars parallel to the long axis of the finger, and 0° and 180° denote bars orthogonal to the long axis of the finger. The preponderance of neurons that are sensitive to orientation and direction of motion respond to contours moving in a direction perpendicular to their orientation.

A hallmark of many visual neurons is that they are sensitive to both direction of motion and stimulus orientation. We ascertained the extent to which neurons exhibited this dual sensitivity by examining their responses to scanned and indented bars. Specifically, we gauged the strength of orientation and of direction tuning in the responses of each neuron in our sample to scanned bars. We found that these neurons spanned the spectrum of tuning properties (Figure 5A). Some neurons (15%) were sensitive to orientation and not direction (Figure 5B); others (36%) were sensitive to direction but not orientation (Figure 5C). A large proportion of neurons (32%), however, were sensitive to both; for example, the neuron shown in Figure 5D responded to a bar oriented perpendicular to the long axis of the finger regardless of whether the bar moved proximal to distal (90°) or distal to proximal (270°), but produced a more robust response in the latter direction than in the former. In area MT, the relative preferred directions and orientations vary widely. However, the preferred direction is often perpendicular to the preferred orientation [17]. We tested whether this was the case for area 1 neurons by comparing the preferred orientation, measured from the responses to indented bars, to the preferred direction (measured from the responses to scanned dot patterns). We found that, indeed, neurons that were sensitive to both orientation and direction exhibited a variety of relative orientation and direction preferences, with a tendency for orthogonal preferences (Figure 5E).

In summary, area 1 comprises a population of neurons that are strongly tuned for stimulus direction and whose tuning is invariant with respect to three major stimulus properties, namely spatial form, speed, or intensity. Furthermore, the responses of these neurons can account for the ability of human observers to discriminate the direction of moving stimuli across a range of conditions. Finally, a large population of neurons is tuned to both stimulus direction and orientation, with the preferred direction predominantly orthogonal to the preferred orientation. These neurons are specialized detectors for moving contours and thus have RF properties that are strongly analogous to those of neurons in primary visual cortex or area MT.

As individual mechanoreceptive afferents are not sensitive to stimulus motion, an explicit representation of motion must emerge at higher processing stages. Computational models have been proposed to describe how the isomorphic representation of the stimulus at the somatosensory periphery is processed to yield information about direction of motion. Direction selectivity has been thought to be conferred by asymmetries in the spatial layout of in-field inhibition (also referred to as replacing or lagging inhibition [2],[18],[19]). However, in-field inhibition is stronger in area 3b than it is in area 1 [19], while neurons in the former exhibit weaker tuning than neurons in the latter. Rather, we propose that direction tuning first emerges in area 3b, produced in part by in-field inhibition and perhaps by mechanisms such as those observed in early visual motion processing (see e.g. [20]). This direction signal is then elaborated in area 1 to yield a more invariant representation of motion direction. Models of the neural mechanisms that produce increasingly invariant motion representations with respect to other stimulus properties at successive processing stages have been developed for the visual system (e.g., [21],[22]). The similarity in the visual and somatosensory representations of stimulus motion suggests that similar mechanisms may be involved in developing these representations in the two modalities [4].

Interestingly, complex processing of motion signals, in some ways analogous to that observed in area MT, occurs in a primary sensory area. Note, however, that area 1 is not strictly a primary somatosensory area [23]. Indeed, thalamocortical projections to area 1 are sparser, target layer III rather than layer IV, and comprise finer fibers than do those to areas 3a and 3b [24]–[27]. Furthermore, neurons in area 1 also receive strong projections from area 3b [28]. Indeed, many neurons in area 1 have larger RFs than do neurons in area 3b and are thought to each receive convergent input from multiple 3b neurons [29]. They are also less linear in the stimulus displacement profile than are their 3b counterparts [3],[19], which may in part account for the invariance of the representation of motion direction they carry with respect to stimulus parameters such as spatial form and speed. Area 1 also comprises a strong representation of stimulus orientation [3] and texture [30], which suggests that it serves other functions and is not a dedicated area for motion processing. The contiguity of form, texture, and motion representations in somatosensory cortex is not surprising given that motion is a hallmark of tactile exploration. Information about motion direction may indeed be necessary to resolve the spatial relationships between stimulus features during scanning [31].

## Materials and Methods

### Stimuli

Stimuli were generated and delivered with a device consisting of 400 independently controlled pins arrayed over a 1 cm^2^ area [11]. This array allows us to generate complex spatio-temporal patterns that simulate the kind of stimulation generated when a finger contacts a surface or object. Bars, scanned dot patterns, and random dot displays at 100% coherence yielded strong motion percepts as reported in pilot psychophysical experiments. Subjects were also able to clearly discern the spatial structure of the bars and the dot patterns (see Text S1 for a discussion of tactile acuity and skin mechanics).

#### Scanned bars

On each trial, a bar was scanned across the skin. The scanning direction was perpendicular to its orientation and ranged from 0° to 337.5° in 22.5° steps (see Figure 1). The amplitude (or depth of indentation) of the bars was 300, 500, or 700 µm; their thickness was 1 mm; and their length was 1 cm. The scanning speed was 40 mm/s and the inter-stimulus interval was 200 ms. Bars were each presented five times in pseudorandom order for a total of 240 trials (16 directions × 3 amplitudes × 5 presentations).

From the neuron's responses to scanned bars, we established its preferred direction (i.e., the direction at which its response was maximal). In a subsequent set of measurements testing the effects of speed on direction selectivity, bars were scanned in the preferred and anti-preferred direction (i.e., bars at the same orientation but moving in opposite directions), as well as in the two orthogonal directions. The amplitude of the bars was 500 µm, their width 1 mm, and their scanning speed 10, 20, 40, or 80 mm/s. Bars were each presented five times in pseudorandom order for a total of 80 trials (4 directions × 4 speeds × 5 presentations).

#### Scanned dot patterns

Individual dots consisted of truncated spheres with a diameter of 2 mm and an amplitude of 500 µm. The dots were arrayed in a diamond lattice and the shortest distance center-to-center between dots was 5.2 mm (see Figure 1B inset). The patterns were scanned at a speed of 40 mm/s in a direction at a 5-degree angle from the orientation of the lattice to minimize repetition in the pattern of dots scanned across the skin. Each pattern was scanned five times in each of 16 directions, ranging from 0° to 337.5° in 22.5° increments. Each stimulus lasted for 1 s with 50 ms on- and off-ramps. Patterns were each presented five times in pseudorandom order with a 100 ms blank interval between each pattern for a total of 80 trials (16 directions × 5 trials).

#### Random dot displays

This paradigm was inspired by visual displays developed by Newsome [12]. The dynamic random display consists of dots that move in the same direction more or less coherently. The strength of the motion signal is determined by the percentage of dots (coherence) that move in a predetermined direction. Each dot persisted for 30 ms before it vanished (including a 15 ms on-ramp and a 15 ms off-ramp) and reappeared as a new dot at a random position. To generate smooth motion, the on-ramp of its reappearance coincided with the off-ramp of its disappearance: the new dot overlapped the existing dot by 15 ms. Because of the limitations imposed by tactile acuity (see Text S1), dots re-appeared at least 2.5 mm away from any other existing dot in the display. Individual dots were hemispheric with a diameter of 2 mm and an amplitude of 450 µm. In any given frame, the density of the display was 5 dots/cm^2^. The percept evoked by a stimulus with 0% coherence is twinkling dots without any global direction of motion. In each frame, each dot reappeared at a location 0.75 mm away from the point at which it vanished (thus moving 0.75 mm in 15 ms, a speed of 50 mm/s). Each dot moved along the predetermined direction of motion with a probability determined by the coherence. Otherwise, the dot reappeared at a random location subject to the constraint described above. In the extreme condition (100% coherence), all the dots moved coherently in the same direction. The direction of motion of the coherently moving dots was the preferred direction of the neuron (as determined from its responses to scanned dot patterns) or one of seven other directions distributed in 45° increments away from the preferred direction. The coherence of the dots was 0%, 14%, 29%, 43%, 57%, 71%, 86%, or 100%. Each stimulus lasted 2.15 s and was followed by a blank interval lasting 100 ms. Random dots displays at each level of coherence were presented five times in each direction in pseudorandom order.

#### Indented bars

This protocol was implemented to gauge the degree to which the responses of individual neurons were tuned for stimulus orientation. On each trial, a bar was indented into the skin at one of eight orientations, ranging from 0° to 157° in steps of 22.5°. Zero degrees corresponded to the axis perpendicular to the long axis of the finger; 90° corresponded to the axis parallel to the long axis of the finger. The amplitude of the bar was 500 µm, its width 1 mm, and its duration 100 ms. The pivot of the bar was either located at the hotspot or was offset relative to the hotspot by 1 to 4 mm in the axis normal to the orientation of the bar. The inter-stimulus interval was 100 ms. Bars were each presented 10 times in pseudorandom order for a total of 720 trials (8 orientations × 9 locations × 10 presentations).

### Neurophysiology

#### Peripheral experiments

All experimental protocols complied with the guidelines of the Johns Hopkins University Animal Care and Use Committee and the National Institutes of Health Guide for the Care and Use of Laboratory Animals. Single unit recordings were made from the ulnar and median nerves of four Rhesus macaques (Macaca mulatta) using standard methods [32]. Standard procedures were used to classify mechanoreceptive afferents according to their responses to step indentations [32],[33].

The point of maximum sensitivity of the afferent (or hotspot) was located on the skin using a handheld probe and then marked with a felt-point pen. The stimulator probe was centered on the point of maximum sensitivity (or hotspot) of the afferent. We recorded from an afferent only if its RF was located on the distal fingerpad of digits 2–5 and if the probe could readily be centered on the RF. We did not record responses from Pacinian (PC) afferents because these have been shown to be highly insensitive to the spatial properties of stimuli presented to their RFs [34].

#### Cortical experiments

Before the microelectrode recordings, surgery was performed to secure a head-holding device and recording chambers to the skull. Surgical anesthesia was induced with ketamine HCl (20 mg/kg, i.m.) and maintained with pentobarbital (10–25 mg kg^−1^ hr^−1^, i.v.). All surgical procedures were performed under sterile conditions and in accordance with the rules and regulations of the Johns Hopkins Animal Care and Use Committee.

Extracellular recordings were made in the postcentral gyri of one hemisphere in each of five macaque monkeys using previously described techniques [35]. The animals were trained to sit in a primate chair with their hands restrained while tactile stimuli were delivered to the distal pads of digits 2, 3, 4, or 5. All recordings were performed with the monkeys in an awake state, which was maintained by offering them liquid rewards at random intervals. On each recording day, a multielectrode microdrive [35] was loaded with seven quartz-coated platinum/tungsten (90/10) electrodes (diameter, 80 µm; tip diameter, 4 µm, impedance 1–3 MΩ at 1,000 Hz). The electrodes were then driven into the cortex until they encountered neurons in area 1 with RFs on the distal fingerpad. A day spent recording from area 1 was typically followed by a day spent recording from area 3b.

When recording from area 3b, the electrodes were driven 2–3 mm below the depth at which neural activity was first recorded. As one descends from the cortical surface through area 1 into area 3b, the RFs progress from the distal to middle, to proximal finger pads, then to the palmar whorls. Within area 3b, the RFs proceed back up the finger, transitioning from proximal to medial and ultimately to distal pads. Because responses from the distal pad were never encountered in the more superficial regions of 3b (where the palmar whorls or proximal pad typically were most responsive), there was never any difficulty distinguishing neurons in area 1 from neurons in area 3b. On every second day of recording, the electrode array was shifted ∼200 µm along the postcentral gyrus until the entire representation of digits 2–5 had been covered. On the third day, we moved the electrodes posterior-laterally to record from area 2. Multi-units in this area had larger RFs and responded to both cutaneous stimulation and joint manipulation, and so were easily distinguishable from their counterparts in area 1.

Recordings were obtained from neurons that met the following criteria: (1) the neuron responded to cutaneous stimulation, (2) action potentials were completely isolated from the background noise, (3) the RF of the neuron included at least one of the distal finger pads on digits 2–5 (only the distal fingerpads of digits 2–5 could be accessed with the stimulator), and (4) the stimulator array could be positioned so that the RF of the neuron was centered on the array. None of the neurons had PC-like properties (i.e., had large RFs and responded to puffs of air).

### Psychophysics

#### Subjects

The subjects were undergraduates at Johns Hopkins University and were paid for their participation. All testing procedures were performed in compliance with the policies and procedures of the Institutional Review Board for Human Use of Johns Hopkins University. Twenty-one subjects (8 males, 13 females) participated in some or all of the psychophysical experiments. In the clockwise-counterclockwise direction discrimination experiment, 15 subjects (7 males, 8 females) were tested with bars, 10 with dot patterns (5 males, 5 females), and 8 with random dot displays (3 males, 5 females). Ten subjects (3 males, 7 females) participated in the left-right discrimination task.

#### Clockwise–counterclockwise direction discrimination

On each trial, the (human) subject was presented with a pair of stimuli (bars, dot patterns, or random dot displays) separated by a 1 s blank interval. The first stimulus, the standard, moved in one of eight directions ranging from 0° to 315° in 45° increments. The second stimulus, the comparison, moved in a direction rotated clockwise or counterclockwise relative to that of the standard by a given angle (ranging from 0° to 50°; see Figure 4). The subject's task was to determine whether the direction of motion of the comparison was rotated clockwise or counterclockwise relative to that of the standard. In each experimental run, standards moving in a given direction were blocked so that the clockwise or counterclockwise decision was trivial to the extent that the veridical directions of the stimuli were perceived. The stimuli were identical to those presented in the neurophysiological experiments except for the presence of a mask: Only pins within a circular area (with a 1 cm diameter) were active to preclude subjects from using positional cues. As was the case in the neurophysiological experiments, the bars and dot patterns were scanned at a speed of 40 mm/s, the random dots moved at a speed of 50 mm/s, the duration of the dot patterns was 1 s, and that of the random dot displays was 2 s (the duration of the bars was determined by the time it took each bar to scan across the display, namely 250 ms). Each stimulus was presented 20 times in pseudorandom order.

#### Left–right direction discrimination

On each trial, the subject was presented with a random dot display with a motion coherence ranging from 0% to 100% spaced in 14% increments. The random dot displays were identical to those used in the neurophysiological experiments. The direction of the coherently moving dots was either 0° (right) or 180° (left). Their task was to indicate whether the dots moved left or right. Each stimulus was presented 20 times in pseudorandom order.

### Analysis

#### Measurement of the response to scanned bars, dot patterns, and random dot displays

Because scanned bars did not overlap a neuron's RF during the entire stimulus interval, steps were taken to ensure that neural responses were measured over the period during which the stimulus was impinging upon the cell's RF. Specifically, the mean spiking rate (impulses per second) evoked by a scanned bar was measured over a 4 mm area centered around the neuron's hotspot.

The mean spiking rates evoked by dot patterns and random dot displays were measured beginning 150 ms after the onset of the stimulus in each trial to exclude responses evoked during the on-ramps.

### Index of Direction Tuning

To gauge the strength of direction tuning in the responses to scanned bars and dot patterns, we used vector strength:
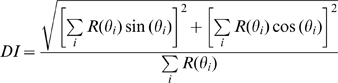
(1)where *R(θ_i_)* is the neuron's response to a stimulus (bar or dot pattern) scanned in direction *θ_i_* [36]. Values of DI ranged from 0, if for example a neuron responded uniformly to all scanning directions, to 1, when a neuron only responded to stimuli scanned in one direction. The statistical reliability of DI was tested using a standard randomization test (*α* = 0.01).

The preferred direction was determined by computing the weighted circular mean:
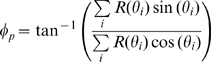
(2)


### Index of Orientation Tuning

We wished to assess the degree to which individual neurons also conveyed information about stimulus orientation. To that end, we computed an orientation selectivity index (OI), analogous to the DI:
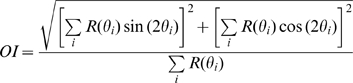
(3)(see above for conventions). Values of OI ranged from 0, if for example a neuron responded uniformly to all orientations, to 1, when a neuron only responded to stimuli at a single orientation. The statistical reliability of OI was tested using a standard randomization test (*α* = 0.01).

The preferred orientation was then determined by computing the weighted circular mean with a moment of 2:
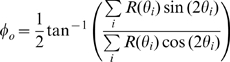
(4)


### Comparing Psychophysics with Neurophysiology

We wished to ascertain the extent to which neural responses could account for our psychophysical data using a standard ideal observer analysis. For each neuron, we randomly sampled, with replacement, from one of the five neural responses evoked by a stimulus moving in its preferred direction and one of the 10 neural responses evoked by a stimulus moving in a direction shifted by |Δ*ϕ*| degrees relative to the preferred direction (there were five presentations of each stimulus and we assumed that ±Δ*ϕ* are equivalent). We repeated this procedure 500 times and computed the proportion of times the response was greater in the preferred direction than when the direction was shifted by Δ*ϕ* for each Δ*ϕ* (ranging from 0° to 180°). Thus, to obtain the relative distributions of neural responses at *ϕ_p_* and *ϕ_p_*±22.5° evoked in a cell whose preferred direction was 90°, we sampled, on each of 500 iterations, one response evoked by a stimulus moving in the proximal-to-distal direction (90°) and one response evoked by a stimulus moving at 67.5° or 112.5°. We then computed the proportion of times the former was larger than the latter. The resulting neurometric functions provide an estimate of how well one could discriminate direction of motion based on the responses of individual neurons.

A similar analysis was performed to compute the neurometric function for random dot displays at various coherence levels: At each coherence level, responses to stimuli at the neuron's preferred direction were compared to responses to stimuli at its anti-preferred direction.

### Fitting Orientation and Direction Tuning Curves

For the purposes of illustration, we fit the data shown in Figure 5B–D with orientation, direction, and combined orientation/direction tuning functions, respectively. For Figure 5B, we used a von Mises function (circular Gaussian) with a moment of 2:
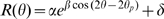
(5)where *R(θ)* is the neuronal response to a bar scanned in direction *θ*, *θ_p_* is the preferred orientation of the neuron, and *α*, *β*, and *δ* are free parameters representing the depth of modulation of the response, the width of tuning for orientation, and the baseline response, respectively. This function denotes orientation tuning. For Figure 5C, we used a von Mises function with a moment of 1, which denotes direction tuning:
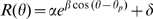
(6)(see above for conventions). Finally, for Figure 5D, we used products of von Mises functions of the form:
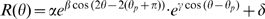
(7)where *α*, *β*, *γ*, and *δ* are free parameters representing the depth of modulation of the response, the width of tuning for orientation, the width of tuning for direction, and the baseline response, respectively. The preferred orientations of the three neurons shown in Figure 5B–D were perpendicular to their preferred directions as reflected in the fitted function (there is only one parameter denoting preference, namely *θ_p_*). We also fit summed von Mises and found that the fits were equivalent but required an additional parameter (because orientation and direction tuning needed to be weighted independently).

## Supporting Information

Figure S1 **Skin mechanics.** (A) Indentation profile for a snapshot of a random dot display with two dots spaced at the minimum allowed distance (the two bottom right dots are spaced 2.5 mm apart). (B) Corresponding strain profile at the depth of the receptor sheet (500 µm), estimated using a continuum mechanics model [2]. As can be seen from the strain profile, the strains elicited by the two adjacent dots are almost completely non-overlapping.
(0.67 MB TIF)

Figure S2 **Response to scanned bars of the most direction selective SA1 fiber in the population.** (A) Raster plot of the afferent response as a function of stimulus direction. (B) Mean rate as a function of stimulus direction (inset: RF of the afferent as measured from its responses to scanned bars). Although this afferent's response was significantly tuned for direction (DI = 0.1), the modulation of its response was weak.
(0.57 MB TIF)

Figure S3 **Effect of amplitude on the responses of mechanoreceptive afferents to scanned bars (red: SA1; cyan: RA afferents).** (A) The strength of the direction tuning was weak across stimulus amplitudes. (B) The preferred direction was not consistent across stimulus amplitudes (only 44% of afferents exhibited preferred directions that differed by less than 45° across the two amplitudes; circular correlation = 0.34, *p*>0.1).
(0.46 MB TIF)

Figure S4 **Direction tuning strength (DI) as a function of speed for neurons that are most strongly tuned at 10 mm/s (A), 20 mm/s (B), 40 mm/s (C), and 80 mm/s (D).** The tuning strength of individual neurons was robustly modulated by scanning speed.
(0.43 MB TIF)

Figure S5 **Median difference thresholds obtained from human psychophysical subjects (cyan) and from individual neurons in area 1 (red) for bars, dot patterns, and random dot displays.** The same data were used to generate this figure and Figure 4 in the main text. Thresholds were obtained by fitting sigmoidal functions to psychometric functions obtained from individual subjects or neurometric functions derived from the responses of individual neurons. The threshold, estimated from the fitted function, was the change in stimulus direction that was discriminated 75% of the time. The direction of motion could be distinguished as well or better based on the responses of individual neurons than it could by humans in a clockwise-counterclockwise task.
(0.35 MB TIF)

Text S1 **Tactile acuity and skin mechanics.**
(0.03 MB DOC)

## Abbreviations

DI: direction selectivity index
OI: orientation selectivity index
PC: Pacinian
RF: receptive field
