# Supplementary figures and images for: Shape Invariant Coding of Motion Direction in Somatosensory Cortex
Source: PLoS Biol. 2010 Feb 2;8(2):e1000305. doi: 10.1371/journal.pbio.1000305 (PMC2814823; doi:10.1371/journal.pbio.1000305)

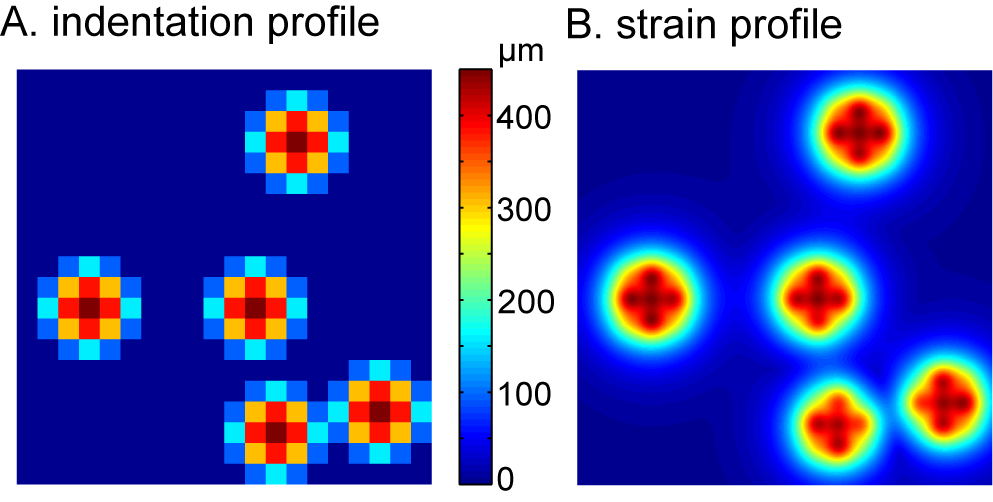

Supplement: Figure S1 — Skin mechanics. (A) Indentation profile for a snapshot of a random dot display with two dots spaced at the minimum allowed distance (the two bottom right dots are spaced 2.5 mm apart). (B) Corresponding strain profile at the depth of the receptor sheet (500 µm), estimated using a continuum mechanics model [2]. As can be seen from the strain profile, the strains elicited by the two adjacent dots are almost completely non-overlapping. (0.67 MB TIF) [file pbio.1000305.s001.tif]

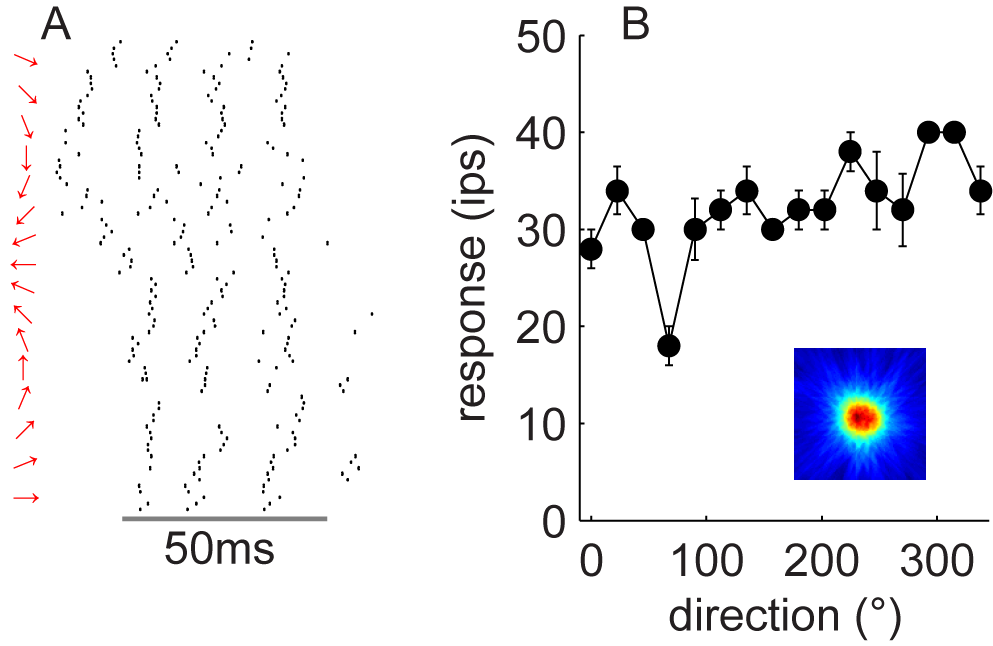

Supplement: Figure S2 — Response to scanned bars of the most direction selective SA1 fiber in the population. (A) Raster plot of the afferent response as a function of stimulus direction. (B) Mean rate as a function of stimulus direction (inset: RF of the afferent as measured from its responses to scanned bars). Although this afferent's response was significantly tuned for direction (DI = 0.1), the modulation of its response was weak. (0.57 MB TIF) [file pbio.1000305.s002.tif]

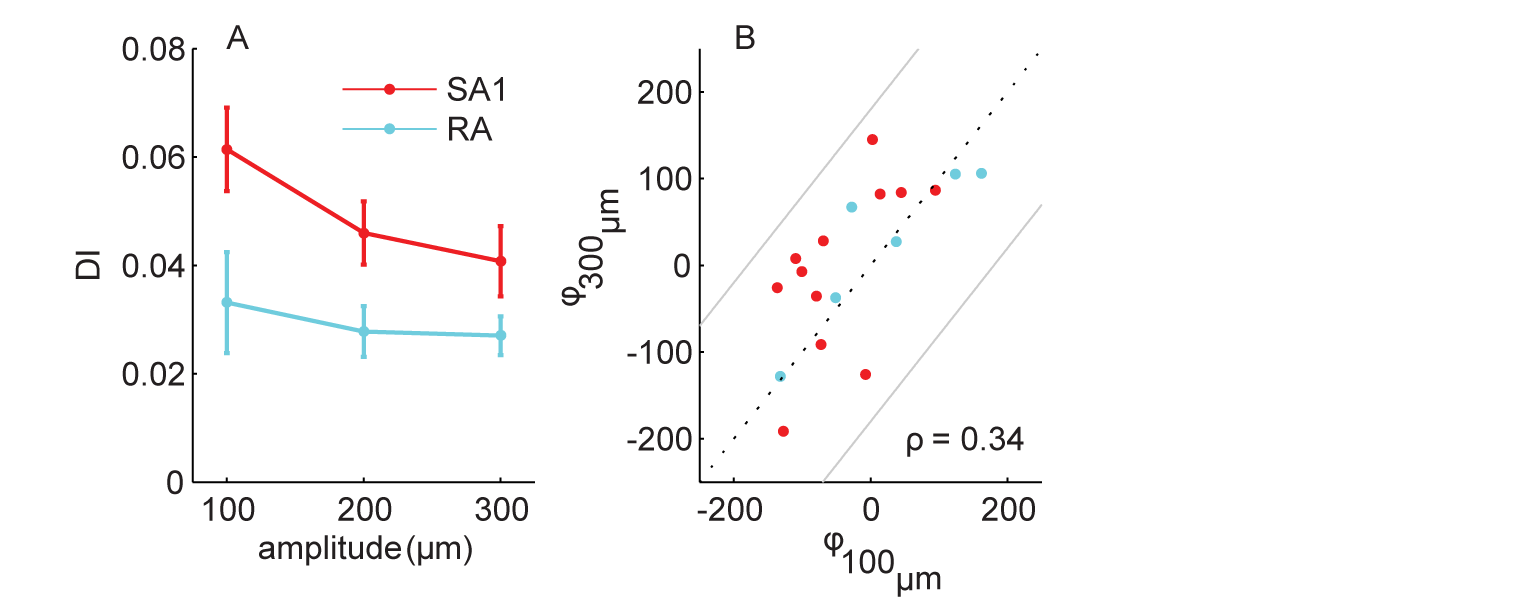

Supplement: Figure S3 — Effect of amplitude on the responses of mechanoreceptive afferents to scanned bars (red: SA1; cyan: RA afferents). (A) The strength of the direction tuning was weak across stimulus amplitudes. (B) The preferred direction was not consistent across stimulus amplitudes (only 44% of afferents exhibited preferred directions that differed by less than 45° across the two amplitudes; circular correlation = 0.34, p>0.1). (0.46 MB TIF) [file pbio.1000305.s003.tif]

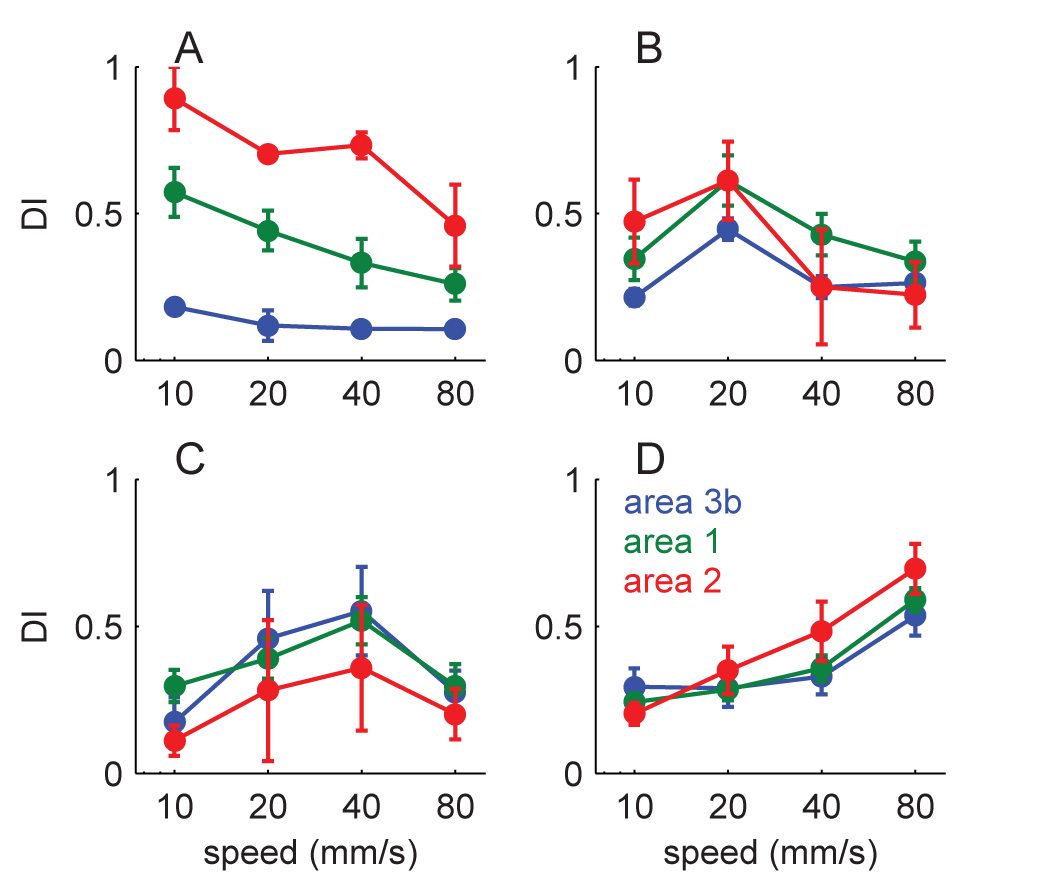

Supplement: Figure S4 — Direction tuning strength (DI) as a function of speed for neurons that are most strongly tuned at 10 mm/s (A), 20 mm/s (B), 40 mm/s (C), and 80 mm/s (D). The tuning strength of individual neurons was robustly modulated by scanning speed. (0.43 MB TIF) [file pbio.1000305.s004.tif]

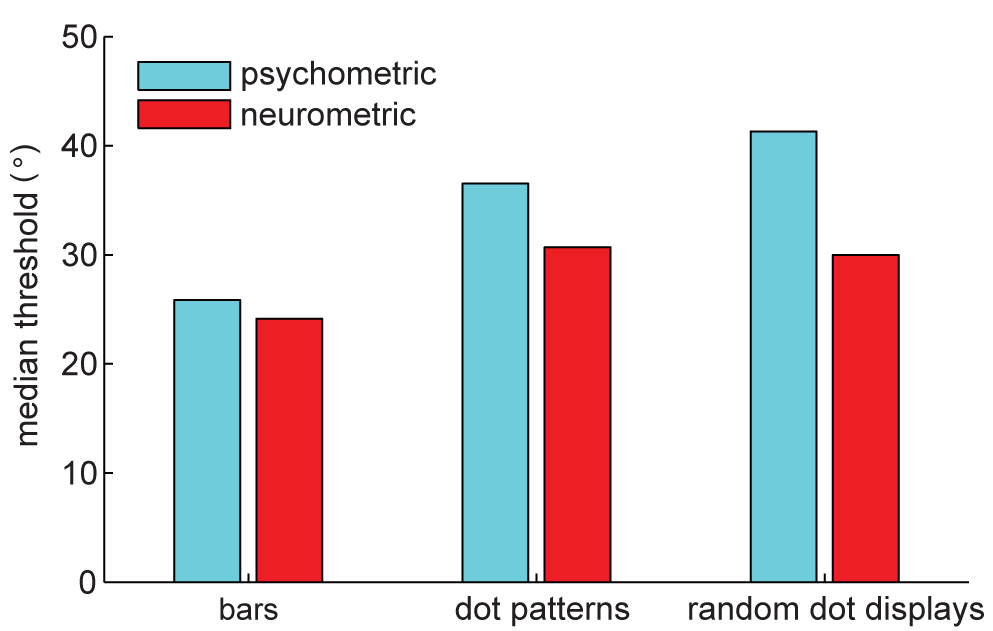

Supplement: Figure S5 — Median difference thresholds obtained from human psychophysical subjects (cyan) and from individual neurons in area 1 (red) for bars, dot patterns, and random dot displays. The same data were used to generate this figure and Figure 4 in the main text. Thresholds were obtained by fitting sigmoidal functions to psychometric functions obtained from individual subjects or neurometric functions derived from the responses of individual neurons. The threshold, estimated from the fitted function, was the change in stimulus direction that was discriminated 75% of the time. The direction of motion could be distinguished as well or better based on the responses of individual neurons than it could by humans in a clockwise-counterclockwise task. (0.35 MB TIF) [file pbio.1000305.s005.tif]
