# Supplementary material for: Shape Invariant Coding of Motion Direction in Somatosensory Cortex
Source: PLoS Biol. 2010 Feb 2;8(2):e1000305. doi: 10.1371/journal.pbio.1000305 (PMC2814823; doi:10.1371/journal.pbio.1000305)
Supplement: Text S1 — Tactile acuity and skin mechanics. (0.03 MB DOC) [file pbio.1000305.s006.doc]

**Text S1**

Tactile acuity and skin mechanics

Forces exerted on the skin surface are distributed through the tissue before they impinge upon individual mechanoreceptors in the skin [1,2]. This spatial blurring, coupled with the limits on spatial acuity imposed by the density of mechanoreceptors, play an important role in determining how spatial patterns indented into the skin are represented at the somatosensory periphery [3]. Accordingly, the stimuli presented in this study were designed to minimize the effects of skin mechanics. Indeed, the spacing between dots was at least 2.5mm, well beyond the spacing between adjacent SA1 afferents, which is about 1mm. Using a continuum mechanics model [2], we verified that adjacent dots were processed independently at the somatosensory periphery (see Figure S1). We verified that adjacent dots were perceived to be distinct in pilot psychophysical experiments.

Reference List

1. Phillips JR, Johnson KO (1981) Tactile spatial resolution: III. A continuum mechanics model of skin predicting mechanoreceptor responses to bars, edges, and gratings. J Neurophysiol 46: 1204-1225.

2. Sripati AP, Bensmaia SJ, Johnson KO (2006) A continuum mechanical model of mechanoreceptive afferent responses to indented spatial patterns. J Neurophysiol 95: 3852-3864.

3. Phillips JR, Johnson KO (1981) Tactile spatial resolution: II. Neural representation of bars, edges, and gratings in monkey primary afferents. J Neurophysiol 46: 1192-1203.
